# Workplace interventions for Finnish nurses: a retrospective document analysis of disciplinary decisions related to substance use

**DOI:** 10.1177/14550725251351702

**Published:** 2025-06-30

**Authors:** Katrimaija Luurila, Mari Kangasniemi, Marja Hult, Arja Häggman-Laitila

**Affiliations:** Department of Nursing Science, Faculty of Health Science, University of Eastern Finland, Kuopio, Finland; Shared Group Services, University of Helsinki and Helsinki University Hospital, Helsinki, Finland; Department of Nursing Science, Faculty of Medicine, University of Turku, Turku, Finland; The Wellbeing Services County of Southwest Finland, Turku, Finland; Department of Nursing Science, Faculty of Medicine, University of Turku, Turku, Finland; Sustainable Well-being South-Eastern Finland University of Applied Sciences, Mikkeli, Finland; Department of Nursing Science, 205537 Faculty of Health Science, University of Eastern Finland, Kuopio, Finland

**Keywords:** employee discipline, nurses, personnel management, substance-related disorders

## Abstract

**Aim:** The study aimed to describe the workplace interventions for registered nurses (RNs) with substance use disorder (SUD) related violations leading to disciplinary actions. **Methods:** A retrospective document analysis of disciplinary decisions related to RNs with SUD (N = 171) from a Finnish regulatory authority. The data were analyzed using descriptive statistics and qualitative methods. **Results:** Substance abuse at the workplace involved intervention actions by a nurse leader and employer with work ability assessment and measures, legislation-based measures for hearing the worker, investigation and handling. Substance abuse services and occupational health services were involved in more than half of incidents. In most of the cases, the RN's contract terminated. **Conclusions:** Further research about the interventions and supervision of healthcare workers with SUD could help clarify protocols and develop measures for the early detection at work places.

## Introduction

There is growing global shortage of nursing workforce (World Health Organization, 2020) which is related to the organizational and managerial, policy-making, educational and individual factors (Tamata & Mohammadnezhad, 2023). It is worth recognizing all of the risks related to nurses health to secure their work ability. Substance use disorder (SUD) is defined as the abuse of various substances (Robinson & Adinoff, 2016) that noticeably influence nurses’ health and safe work performance (Kunyk et al., 2016). At the workplace, SUD can be detected through diverse behavioral and physical signs (Alunni-Kinkle, 2015), along with other indicators such as medication ambiguities (Trinkoff et al., 2021). The prevalence of SUD among nurses has been reported to be similar to the level seen in the general population (Kunyk, 2015). Nevertheless, other reports have stated that the prevalence among nurses can vary by 5‒20% based on the method of estimation (Mumba & Kraemer, 2019). Fields with stressful work and easy access to medicines have been mentioned as a risk factor for more pronounced substance use (Darbro & Malliarakis, 2012). Lately, nurses in long-term care and home health care, fields with lower accountability and supervision than other areas of health care, have shown high rates of SUD and impaired practice caused by substance abuse (Mumba et al., 2019; Trinkoff et al., 2022). Little is known about workplace interventions used for nurses with SUD.

### Background

Previous studies have described violations by nurses with SUD as intentional or unintentional mistakes, errors and misconduct caused by intoxication or controlled substances (CS). SUD-related violations have led to serious patient safety issues and self-harm risks for nurses (Brummond et al., 2017; Crigger & Godfrey, 2014; Pohjanoksa et al., 2019), as well as criminal cases (New, 2014). Individual nurses are responsible for their ability to work (ICN, 2021), but, when affected by SUD, a person faces a risk of losing control over their choices and behavior (Burda, 2020). A review of the disciplinary decisions from a regulatory authority highlighted that SUD is one of the most common primary reasons for nurses’ unethical conduct (Papinaho et al., 2022a). In those cases, disciplinary decisions have led to warnings and the interruption of a professional career via licensure restriction, suspension, or revocation (Papinaho et al., 2022b).

Each nurse has an essential frontline role in detecting misbehavior or wrongdoing by their colleagues, for instance SUD-related offenses, and is responsible for reporting any risk to patient safety or the work environment to their supervisor (ICN, 2021; Kunyk, 2015). Nurses can recognize colleagues’ unsafe behavior and cues as indications of unsafe practice (Blair et al., 2016, 2021) but nursing peers have demonstrated a lack of confidence in their ability to identify colleagues with SUD (Trinkoff et al., 2021). Nurses need response strategies to demonstrate professional values under challenging situations (Numminen et al., 2017). Nurses could benefit from educational initiatives covering signs of SUD and the development of a culture in which everyone feels accountable to address their colleagues’ SUD-related problems (Cares et al., 2015; Trinkoff et al., 2021).

Nurse leaders have a crucial role in establishing workplace practices and norms to promote patient safety and encourage open communication among staff (Seo & Lee, 2022). This includes intervening when they notice misbehavior of wrongdoing by nurses with SUD (Kunyk, 2015). Nurse leaders have previously stated that intervening is ethically challenging because of the sensitive and personal nature of SUD problems (Ganz et al., 2015). In addition, this action requires the ability to assess and observe situations (Cadiz et al., 2015; Taylor, 2020), as well as moral courage to talk about a sensitive situation (Laukkanen et al., 2016; Rasoal et al., 2017). In addition, a nurse leader is responsible for considering whether a violation or offense by a nurse requires organizational programs for investigation (Brummond et al., 2017; Griffith et al., 2021) or occupational health services (OHS) intervention. Finally, in the case of a serious patient safety risk, nurse leaders have the duty to proceed to a regulatory authority investigation.

Despite national and cultural differences in the processes for intervening and investigating wrongdoing by nurses with SUD, there is shared interest in focusing on monitoring (Russell, 2020) and guidelines for procedures related to SUD offences (Smiley & Reneau, 2020). Previous studies of nurses with SUD have focused on the care violations and the associated disciplinary procedures (Papinaho et al., 2019, 2022b) as well as the characteristics of nurses with SUD (Luurila et al., 2023) and the existing alternative-to-discipline programs (i.e. ADPs) (Pace et al., 2020; Russell, 2020). Most of this previous research in the past decade has been conducted in North America (Cares et al., 2015; Kunyk, 2015; Monroe et al., 2013; Mumba et al., 2019; Ross et al., 2018; Trinkoff et al., 2022).

Only a few studies have been conducted in Europe. In Nordic countries, nurses’ substance use has been studied among other healthcare professionals (Edvardsen et al., 2014; Virtanen et al., 2012). Nurses only have been studied, for example, in the UK (Pezaro et al., 2022) by focusing on the perceptions of problematic substance use and its influences on mental and physical health of midwives. As such, there is limited knowledge about workplace interventions for nurses with SUD; this information would be critical to developing early interventions and ethical practices at the workplace. More qualitative research is needed to better understand which procedures are useful and effective for maintain work ability.

The present study aimed to describe workplace interventions for registered nurses (RNs) with SUD related violations which led to regulatory authority disciplinary actions in Finland. The research questions were:

1. How was the detection of substance use carried out?
2. Which actions did the intervention process include?
3. Which parties were involved in the intervention process?
4. What were the consequences of the intervention process?

## Methods

### Study design

This study employed a descriptive, retrospective document analysis of disciplinary decisions against RNs with SUD issued by the Finnish national regulatory authority. The data were analyzed using descriptive statistics and qualitative methods (Moilanen et al., 2022).

### Research context

In Finland, the national regulatory authority is organized as a Supervisory Board, which is a part of the Ministry of Social Affairs and Health. The Board makes disciplinary decisions on healthcare professionals who have seriously threatened patient safety and violated professional legislation. The sanctions include temporary, permanent or indefinite decisions that restrict, suspend or remove a nurse's right to practice; a healthcare professional can also receive a written warning (Act on Health Care Professionals, 1994). The Supervisory Board comprises six members who represent the legal, medical and social care fields, as well as a representative of the profession that is under investigation (National Supervisory Authority for Welfare and Health, 2025). When making a decision, the Supervisory Board will carry out an investigation, including case notifications, reports from the workplace and occupational healthcare, and medication records regarding the RN under investigation. The RN's professional capacity, state of health and professional skills will also be reviewed, and the RN under investigation has a right to provide their own explanation of the issue (Act on Health Care Professionals, 1994).

### Data collection

In this study, disciplinary decisions by the Supervisory Board representing the national regulatory authority against registered nurses with SUD were collected from 1 January 2007 to 31 December 2016. We included disciplinary decisions which focused on: (1) registered nurses and (2) their cases related to SUD. The disciplinary decisions were in paper format, and included the complaint or notification by the complainant, investigation documents of the Supervisory Board and the decision paper. The number of investigation documents per RN varied between 1 and 47, with a mean of 14 documents. In this study, we used the information provided on the decision paper and, when necessary, the other investigation documents were used to clarify the decision paper.

We developed an electronic extraction matrix for data collection (Moilanen et al., 2022). The matrix was based on previous research (Cares et al., 2015; Kunyk, 2015; Wright et al., 2012) and a consultation of the disciplinary authorities. The extraction matrix included 34 items concerning the background information of the RN under investigation, any previous investigation history, the purpose and complainant of the current investigation, along with the detection, interventions and consequences of substance abuse. The items included both numeric (e.g., age and length of working time) and open-ended questions (e.g., description of detection of SUD). The structure and content of the extraction matrix was tested using an initial set of 23 Supervisory Board decisions, after which it was revised and used for the entire data set.

Information related to each disciplinary decision was manually entered into the electronic extraction matrix. If a RN had received several decisions, information covering all of these decisions was collected as one record. Into the extraction matrix, 324 decisions regarding 204 RNs were entered by four researchers (KL, OP, MT, MK)*.* Of these, 171 (83.8%) RNs had decisions related to SUD, and were included in the study.

### Data analysis

The quantitative analysis was used to answer the research questions. The expressions in the decision documents were extracted to the matrix, reduced and condensed into variables as numeric values (Moilanen et al., 2022). We carried out quantitative descriptive analyses and present the results as frequencies and percents. SPSS version 27.0 (IBM Corp., Armonk, NY, USA) was used to perform the analyses. Research questions 2 and 4 were addressed also with the qualitative analysis which followed the principles of content analysis (Vaismoradi et al., 2013). First, we collected all of the relevant expressions from the open ended data with a phrase being the analytical unit. Next, the expressions were coded based on content, and grouped accordingly into categories and subcategories (Moilanen et al., 2022; Vaismoradi et al., 2013). We have illuminated the results for every research question by autent examples of the data in extraction matrix.

### Ethical considerations

The study was conducted in accordance with Finnish legislation (Act on the Openess of Government Activities, 1999; Data Protection Act, 2018), and ethical principles of research were followed (ALLEA, 2017). An ethical review statement was not needed for the type of documentary data collected in this study (Finnish National Board on Research Integrity, 2019) and permission for data collection was granted by the National Supervisory Authority for Welfare and Health. All of the researchers involved in data collection followed the non-disclosure agreement and research-related data protection guidelines issued by the Supervisory Authority. Because the collected material was confidential and sensitive, it was only processed in situ at the premises of the regulatory agency. A representative of the supervisory authority collected manually decision files from their archive and brought them to the researchers for analysis.

Data for the electronic extraction sheet were selected and written on a computer, and gathered without any identification of the RNs (Moilanen et al., 2022). Appropriate and secure curation of the data and research materials was ensured. The encrypted external storage device, which was used to store the collected data, was kept in a locked state. Disposal of the data followed Supervisory Authority's instructions (ALLEA, 2017).

## Results

### Characteristics of the disciplined nurses

Registered nurses with disciplinary processes had an average age of 43 years, with slightly over half being under 45 years of age. Most were women who worked in the healthcare sector. The nurses had been at their current workplace for an average of 5.3 years, with over half of the nurses having worked in the same workplace for over 1 year. Medicines were involved in three-quarters of the cases, whereas alcohol was involved in half of the cases. Almost half of the nurses were diagnosed with mental health disorder and SUD (Table 1).

**Table 1.** Background characteristics of the studied registered nurses (*N* = 171).

	Mean (SD)	*N*	%
Age (years)	42.9 (8.7)		
<45		91	53.2
≥45		78	45.6
*Missing documentation*		2	0.2
Gender			
Women		139	81.3
Men		32	18.7
Working position			
Nurse		146	85.4
Head nurse		14	8.2
*Missing documentation*		11	6.4
Sector			
Health care		113	66.1
Social services		45	26.3
*Missing documentation*		13	7.6
Working history, years	5.3 (7.8)		
<1		63	36.8
≥1		99	57.9
*Missing documentation*		9	5.3
Substance used			
Alcohol		43	25.1
Medicines		89	50.9
Alcohol and medicines		28	17.5
Drugs, alcohol and/or medicines		11	6.5
Diagnoses			
Mental health disorder		41	24.0
SUD^a^		18	10.5
Mental health and SUD^a^		78	45.6
Neurological condition		3	1.8
Musculoskeletal condition		6	3.5
*Missing documentation*		25	14.6

a SUD = Substance use Disorder.

### Detection of substance abuse

Substance abuse at the workplace by RNs was detected in third of the cases based on documentation in medicine cards and on alcohol and drug testing, the second most common method for detecting substance abuse. In almost half of the cases, the intervener was a nurse leader (ward nurse or head nurse), while a nurse peer was the intervener in a third of cases. In a few cases, a patient of the nurse committing the violation, or their relative, was the intervener (Table 2). According to the qualitative descriptions, nurse peers provided frontline prevention for working while intoxicated by sending a nurse home or to acute care. Nurse peers, patients or the patient's relatives informed the RN's superior about drug theft, ambiguities in drug recording or increased drug consumption. They detected drug use or intoxication, groggy or strange behavior, and/or reduced work ability. Interventions were decribed as follows:

The nurse leader noticed that the nurse [under investigation] was confused, walked unsteadily and wobbly, behaved inappropriately and seemed to be intoxicated. The nurse leader referred the nurse to health services for an assessment of work ability and blood and urine tests. (Decision 138)

After coming to work, the nurse [under investigation] seemed to behave strangely and smelled of alcohol. Nurse peers asked the nurse leader to check the situation, because the nurse's [under investigation] speech was indistinct. Alcohol testing verified intoxication. (Decision 89)

**Table 2.** Intervening to nurses’ SUD at work places (*N* = 171).

	*n*	%
Method of detection		
Ambiguities in documentation	59	34.5
Alcohol and drug testing	55	32.2
Sensory finding	42	24.6
*Missing documentation*	15	8.7
Intervening person		
Nurse leader	84	49.1
Nurse peer	51	29.8
Patient or relative of the patient	3	1.8
*Missing documentation*	33	19.3
Parties involved in the intervention process^a^		
Substance abuse services	106	62.0
Occupational health services	89	52.0
Psychiatric services	60	35.1
Substance abuse team of the workplace	36	21.1
Social services	13	7.6
Consequences of interventions^a^		
Dismissal	83	48.5
Resignation	44	25.7
Remark or warning	40	23.4
Limitation of work tasks	21	12.3

a Several mentions per nurse possible.

### Actions taken during the intervention process

Actions taken during the intervention process for nurses’ SUD were described in quantitative data (*N* = 171) and it was complemented with qualitative data concerning 155 nurses. The described actions supplemented the measures initially implemented by the nurse leader or employer. Based on the documents 61% of nurses have had work ability assessments and measures. These actions included collaboration with occupational health services, supervisor–subordinate discussions, guidance to treatment and rehabilitation services (Table 3). The collaboration with occupational health services included tripartite negotiations which were taken to assess a nurse's work ability when verifying an acute situation or to gain guidance for further treatment after an acute situation. The tripartite negotiations involved the nurse, nurse leader and occupational health representative. In these negotiations treatment plans and contracts for SUD were agreed. Collaboration with OHS was described as follows:

The nurse leader had to remove nurse [under investigation] from the workplace due to suspicion of drug use. A tripartite meeting was held with the occupational health service, drug tests were performed, and a positive result was seen. Referral to mental health and substance abuse treatment was made through occupational health service. (Decision 161)

**Table 3.** Actions^a^ taken during the intervention process for nurse with SUD based on quantitative (*N* = 171) and qualitative (*N* = 155) data.

	*n*	%
Work ability assessment and measures (*N* = 171)	104	60.8
Collaboration with occupational health services (*N* = 155)	84	54.2
Supervisor−subordinate discussions (*N* = 155)	67	43.2
Guidance to treatment (*N* = 171)	70	40.9
Guidance to rehabilitation services (*N* = 171)	37	21.6
Legislation based measures for hearing the worker (*N* = 155)	48	31.0
Investigation and handling (*N* = 155)	27	17.5
Criminal report to the police (*N* = 155)	21	13.6
Work community meeting (*N* = 155)	6	3.9

a Several mentions per nurse possible.

Supervisor–subordinate discussions were organized at early phases of the intervention, and comprised prompt chats and deeper discussions according to the early intervention model or treatment guidance model (Table 3). In the case of intoxication, discussions usually took place the day after. Discussions were described as follows:

The nurse leader held an early intervention discussion due to strange behavior and possible substance abuse by the nurse [under investigation]. (Decision 39)

The nurse [under investigation] was removed from the workplace due to intoxication. The discussion was held according to the referral to treatment model. (Decision 87)

Workplaces guided 41% of offending nurses to treatment and 4% of the nurses sought treatment themselves. One-fifth of the nurses were guided to rehabilitation services. One-fifth of the nurses were committed to care.

The second most common type of action described for the intervention process was legislation-based measures for hearing the worker (Table 3). Hearing was related to the procedure for issuing a warning or terminating an employment relationship. The stakeholders of hearings consisted of representatives from the employing organization (nurse leader, chief nurse, manager, personnel administration or safety manager) and the support parties for the nurse under investigation (work safety commissioner, trustee, assisting lawyer or colleague). Hearing was described as follows:

After the drug theft incident, a hearing was held with the branch manager, nurse leader, personnel manager, the organization's lawyer, and the nurse [under investigation], with a support person present. The nurse [under investigation] was notified that the employment relationship will be terminated. (Decision 156)

The third most commonly described action of the intervention process was investigating and handling (Table 3). Criminal reporting to the police was mainly carried out in the cases of drug theft and in the cases of intoxication, when there was assumed drunk driving. The work community meetings were arranged related to the investigation of drug theft and handling the incident with the staff. These two actions were described as follows:

As a result of the investigation related to loss of medicines, the nurse [under investigation] was given a written warning and a criminal report was filed with the police. (Decision 18)

The loss of drugs was brought up in a meeting at the workplace, which included the chief physician, nurse leader and nurse peers. (Decision 81)

### Parties involving in the intervention processes

Different parties from outside of the nurse's work unit were involved in the intervention processes, with more than one party often involved in the process (Table 2). In most cases, substance abuse services were involved. In addition, OHS were involved in half of the interventions, while psychiatric services were involved in the third of the interventions. The substance abuse team from the workplace was involved in the fifth of interventions, while social services were involved in some of the interventions. Involvement of external parties was described as follows:

In the follow-up sessions of the occupational rehabilitation nurse [under investigation], the nurse from the substance abuse service, nurse leader, head nurse, occupational health nurse and shop steward were present. (Decision 91)

### Consequences of the intervention processes

The intervention process had various outcomes, and one nurse could face multiple consequences (Table 2). One-fifth of the nurses received a warning or remark, while a minority had the breadth of their work tasks reduced following the intervention process. The warning given at the hearing was most often a written warning. When a warning procedure was described, employment was often terminated after the first or second warning. Consequences were described as follows:

Due to intoxication, the nurse [under investigation] was given a written warning. The nurse [under investigation] was directed to the occupational safety and health service, which nurse [under investigation] refused and received another written warning. The nurse [under investigation] did not show up for work anymore. The employer terminated the employment relationship due to the refusal of referral to treatment, prior warnings and unauthorized absence. (Decision 147)

Limitation of work tasks was described as the removal of drug administration licenses and the limitation of medical treatment outside of work duties. The reorganization of tasks or changing work tasks was also described. Prohibition of work was described when the violation was caused by intoxication. Limitation and prohibition of work tasks were described as follows:

Due to the suspicion of drug theft, the nurse [under investigation] was interviewed. The nurse [under investigation] was directed to other tasks that did not include the implementation of drug treatment. (Decision 97)

The nurse [under investigation] arrived at work smelling of alcohol. The occupational health nurse carried out a breathalyzer test for the nurse [under investigation] and it was positive. The nurse [under investigation] was removed from the workplace. (Decision 96)

At the end of the intervention process, the employment relationship ended for three-quarters of nurses, almost half were dismissed and one-quarter resigned (Table 2). Termination of the employment relationship was an immediate consequence at end of the intervention process. In cases of intoxication, the employer terminated the employment relationship after the employee in question was intoxicated at work another time. In these cases, the procedures for a remark, warning and referral to treatment had already been used. In cases of drug theft, termination of the employment relationship was also described as immediate, especially when the theft had been reported to the police. The nurse's own resignation was described happening shortly after being found to be intoxicated, after the first hearing, or after several warnings and request to get treatment. Termination of work was described as follows:

The employer had to terminate the nurse's [under investigation] employment contract due to repeated drug diversion, working under the influence medicines and endangering patient safety, as well as violation of the treatment contract. (Decision 148)

## Discussion

The present study provides insight from the Nordic context about workplace interventions for RNs with SUD which have led to regulatory authority disciplinary actions. Most of the previous research into disciplinary procedures resulting from nurses problems with SUD has been conducted in North America. During the research, various elements of the intervention process were compiled from official documents concerning disciplinary decisions by the Finnish national authority.

Disciplinary processes for nurses with SUD were initiated at the workplace when there was a suspicion of substance abuse based on smell, sight or hearing, or a noticeable change in a colleague's work performance. Alcohol and drug testing was used in a third of cases to confirm the initial observation. Drug diversion was also identified as a result of ambiguities in drug records; this was mentioned in a third of the decisions. These signs have previously been described as being worthy of attention for identifying nurses with SUD (Cadiz et al., 2015; Cares et al., 2015; Griffith et al., 2021). Impairment caused by substance use has an established effect on occupational and patient safety, and thus must be noticed as early as possible. Nurses’ awareness of SUD as a disease, as well as the ability to detect and intervene early enough, should be incorporated into the nursing education curriculum (Salani et al., 2022). In addition, all healthcare staff should receive complementary education to promote the early identification of substance use and decrease stigmatization. For example, previous studies have identified that physicians also face stigma because of SUD (Srivastava, 2018; Wilson et al., 2022) and this may cause hiding the problem and prevent early intervention.

In a third of the cases, nurse peers intervened when noticing problems caused by nurses with SUD. Nurse peers recognized the signs, intervened and reported their observations to the nurse leader. According to previous research, colleagues feel that the supervisor is the primary person they should report unsafe practices to (Bettinardi-Angres & Bologeorges, 2011; Blair et al., 2021) or share instances of any suspicions of substance abuse at the workplace (Trinkoff et al., 2021). The low number of interventions by peers is in line with what has been reported in previous studies; more specifically, nurses feel that it is challenging to intervene or whistleblow even when they are aware that the behavior of a colleague could harm others (Pohjanoksa et al., 2019; Trinkoff et al., 2021). Fear of personal consequences (as the whistleblower) and repercussions for their colleague have been identified as obstacles to reporting (Wiisak et al., 2023). On the other hand, it has been reported that less than half of nurses with SUD feel that their colleagues could have recognized their problem earlier (Cares et al., 2015). This could be explained by a nurse's ability to cover the telltale signs of substance abuse because nurses know the adverse events associated with substance abuse or a lack of awareness among peers. There should be strategies and guidelines for nurses how to intervene on collegial way to their peer's unsafe practices and reporting systems that protect reporters from repercussions (Blair et al., 2022). In healthcare workplaces, the frontline role of nurse peers in identifying SUD should be acknowledged, and active measures should be implemented to support this.

In this study, nurse leaders were the primary intervener in half of the cases. This is in line with what has been reported in previous studies (Kunyk, 2015) and agrees with a nurse leader's professional duties (Alunni-Kinkle, 2015). According to this study, the nurse leaders used diverse administrative protocols, early intervention models and the organization's substance abuse program when discussing the violation with nurses affected by SUD. At the organizational level, preventing nurses’ SUD-related violations could be ensured through the maintenance of comprehensive prevention programs for the diversion of CS and substance abuse. These programs should include core administrative elements, system-level controls and provider-level control (Brummond et al., 2017). In clinical practice, rounds focusing on the risk of medicine diversion have been found to provide information on deficiencies that need to be corrected (New, 2014). In addition, nurse leaders need the ethical competence and courage to have serious discussions with employees who have issues with substance abuse (Aitamaa et al., 2019). Nurse leaders need detailed organizational protocols for intervening in the case of substance abuse issues among nurses at the workplace; these protocols could involve constant education for how to engage with nurses with SUD, along with the possibility to obtain organized support when handling difficult ethical issues.

According to the results of this study, a supervisor–subordinate discussion was carried out right after detection of a RN's intoxication or diversion of CS. Discussion were based on the attentions and evidence from detection regarding nurses decreased ability to work. This is in line with previous guidance; more specifically, adequate evidence and information about the situation should be gathered prior to any interview with the nurse (Griffith et al., 2021). A nurse manager's discussions with nurses has been identified as the most useful method in solving ethical problems (Aitamaa et al., 2019). However, more knowledge about the content of these discussions is needed to develop progressive and confidential models to support nurses with SUD. Hearing is the legal and ethical right of the nurse with SUD and gives an opportunity to present explanation for the situation and should be ensured in organizational protocols.

Drug diversion and driving under the influence (DUI) were the most common reasons for why employers contacted the police. Although DUI was found to be the most common criminal conviction related to nurses’ disciplinary actions (Zhong & Martin, 2022), it was only mentioned a couple of times in this study as a reason for contacting the police. This was due to the documents, which described mainly workplace related violations. Also, the current practices related to intoxication at work (i.e., calling the intoxicated nurse a ride or escorting the nurse home) may prevent DUI (New, 2014). Committing crime related to SUD is found to be risk factor for higher recidivism rates (Zhong & Martin, 2022). Hence, the results presented here and in prior research highlight the importance of pre-employment screening by means of looking at a potential employee's clinical employment history (New, 2014). The organization's clear policy for the nurse leaders for pre-employment licensure background checks following national regulation and the necessary procedure instructions must be ensured for to prevent recidivism but also to enable employment if given restrictions have been removed.

In most cases, substance abuse services were involved in the intervention process, and over half of the nurses already had diagnoses of mental health disorders and SUD or SUD alone. In half of the cases, OHS were involved in the intervention process. Although access to the OHS is a legal right of employees (Occupational Health Care Act, 2001; Reho et al., 2021), the number of nurses that the service actually reaches is nevertheless small. This may be influenced by missing information in the decision documents. The substance abuse stigma might influence intention to seek help from OHS because of fear of judgement and legal actions (Fetterhoff, 2023). The involvement of OHS in nurses’ SUD-related violations should be clarified in organizational policies; furthermore, there should be the possibility for nurses with SUD problems to receive individualized, evidence-based treatment options (Ross et al., 2020). In the future, the realization of the rights and protection of nurses must be studied, and information produced used to strengthen those in these processes.

Almost a quarter of RNs received a remark or warning after being intoxicated at the workplace or after diverting drugs. In comparison, previous studies only provide scarce descriptions of the warning procedures that are used in the clinical work environment. Further research is needed about the methods used in organizational supervision at the workplaces’ disciplinary processes to develop effective support for employees SUD related behavior.

One out of 10 of disciplined nurses with SUD got delimitation of work tasks at the workplace. Because most cases were related to drug diversion, the nurses experienced severe restrictions in their ability to carry out medical treatment. Easy access to drugs has already been recognized as one of the main risks to nurses illegally using CS, however, other structural factors such as culture of nursing profession, treatment policies and challenges in work conditions should also be considered (Ross et al., 2018). Research is needed on what kind of support in addition of the limitation of work tasks is needed for effectively to prevent the renewal of drug abuse at the workplace.

Over the half of the RNs involved in the substance abuse violations were dismissed from their job or resigned themselves. In some cases, the supervisors followed guidelines in which an employment relationship can be terminated immediately if the reason is severe enough (e.g., intoxication and/or using intoxicants at the workplace, neglecting a work obligation despite a warning or jeopardizing work safety) (Employment Contracts Act, 2001). According to Zhong and Thomas (2012), a nurse's job history, which will include termination of employment by an employer, is associated with a risk of additional violations and should be noticed by supervisors during recruitment. The nurses with SUD should also find treatment for their problems instead of changing the workplace (Zhong & Thomas, 2012). The early recognition of SUD among nurses is crucial to avoiding the premature termination of careers. At the end of the employment relationship, it must be ensured that treatment facilities cover a nurse with SUD, although it might be challenging with self-dismissals. An important research topic for the future would be the effects of RNs’ SUD-related dismissals, with supervisory authority documents and/or interviews providing empirical evidence.

### Limitations and strengths

The limitations of this study are related to the nature of the analyzed data, data collection, and analysis of qualitative data (Moilanen et al., 2022). The documentation related to decisions were not made for research purposes. As such, the scrutinized documentation was prepared by various officials, was not strictly structured and included an enormous amount of information on each research subject. This data diversity made it challenging for the researchers to select relevant content for the extraction matrix. However, the data, which contained information from various legally eligible documents, demonstrated good reliability.

The involvement of several data collectors may have affected the reliability of the data, especially when qualitative descriptions of intervention processes were gathered. The contents of different interventions were described in various ways in the decision documents. The use of legislation-based documents limits the possibility for rich descriptions of empirical reality and not all of the events of the intervention process will have the detailed information researchers were hoping for. (Moilanen et al., 2022.) However, joint discussions between data collectors were used to strengthen the consistency of data collection and ensure that all decisions on the content were concurrent. Qualitative analysis was secured with and guided by the extraction matrix and analysis units collected according to the matrix (Moilanen et al., 2022).

## Conclusions

The present study has produced new knowledge from Finland concerning workplace interventions for nurses with SUD-related violations which led to regulatory authority disciplinary actions. Substance abuse among nurses was detected from medicine records, via observations by peers, or through alcohol and/or drug testing. The intervener was most often a nurse leader or nurse peer. The intervention process involved the following actions: work ability assessment and measures; legislation-based measures for hearing the worker; and investigation and handling. The documentation revealed that the intervention process usually involved several parties outside of the work unit (e.g., OHS). The consequences of SUD-related violations were remarks or warnings, limitation of work tasks and termination of the employment relationship. Althought the results cannot be generalized, the present research highlights that the field of SUD among nurses and interventions at the work place requires more attention because different parts of the research identified new study questions that remain unanswered. Further research about the interventions and supervision of healthcare workers with SUD could help clarify protocols and develop measures for the early detection at work places.

To support nurses’ work ability in times of nurse shortage, further research into SUD-related violations could be used to develop the protocols for interventions and protect the rights of nurses with SUD. It is important that nurse leaders and nurse peers are aware of the signs of SUD and know how to intervene early in a way that is consistent with the current legislation. Moreover, each organization should periodically check whether the current guidelines for interventions are clear to understand and easy for any employee to implement. Each employee should also have the ability to take part in training about SUD-related protocols and interventions. Lastly, nursing education should sufficiently cover SUD-related issues in the clinical environment, and these aspects should be periodically covered in complementary education for employees.
